# Dermatology Publications in Saudi Arabia: A Fast-Growing Pattern

**DOI:** 10.7759/cureus.38006

**Published:** 2023-04-23

**Authors:** Abdulrahman I Alfawzan, Riam S Alkhamis, Dalal Alshagha, Lamia AlAkrash, Nouf Almohanna

**Affiliations:** 1 Dermatology, King Abdulaziz Medical City, Riyadh, SAU; 2 Dermatology, Unaizah College of Medicine and Medical Sciences, Al-Qassim, SAU; 3 Dermatology, King Fahad Medical City, Riyadh, SAU

**Keywords:** scopus, web of science, wos, saudi arabia., kingdom of saudi arabia (ksa), bibliometrics, bibliometric analysis, publications, dermatology

## Abstract

Bibliometric analysis provides an accurate report of the quantity and quality of research affiliated with a specific country. We aimed to use bibliometric analysis to evaluate previously published dermatology-related studies from Saudi Arabia (SA). We conducted a retrospective, cross-sectional bibliometric analysis using the Web of Science (WoS) and Scopus databases to retrieve all SA-affiliated dermatology research from the databases’ respective dates of conception to July 9, 2021.

The number of publications was determined by the total number of articles, the number of times an article was cited, publishing journals, and affiliated institutions. The Hirsch index (h-index) was used to describe the quality of the articles. In total, SA-affiliated dermatologists published 1,319 articles in WoS and Scopus. Approximately half (n=603) of these articles were published in the past six years. According to WoS, the total number of citations was 9,285 with more than half of all citations also occurring in the past six years. The International Journal of Dermatology was associated with the highest number of publications, followed by the Journal of the American Academy of Dermatology. SA had the second-highest number of publications in the Arab world.

Our area has recently experienced rapid growth in dermatology publications. We encourage the use of data from the current study to identify the strengths and weaknesses of such publications, to direct researchers and funds to enhance the national growth of dermatology research, and to conduct periodic bibliometric analyses with the aim of assessing the quality and quantity of SA-affiliated publications over time.

## Introduction and background

Saudi Arabia (SA) is the largest country in Western Asia, with a land area of more than two million kilometers and geographic, demographic, and economic advantages. Its government continues to value research and the development of research centers. There are 68 universities and degree-granting institutes in SA, and the educational budget reached its highest-ever level ($56.56 billion) for the year 2014 [1,2]. The government has introduced the National Plan for Science, Technology, and Innovation to encourage academic work and research. This program aims to provide the infrastructure needed to develop SA as an internationally advanced knowledge-based economy with competitive science and technology programs [3].

Bibliometrics is defined as using mathematical techniques to investigate publishing and communication patterns in the distribution of information [4]. This method has improved in terms of complexity, accessibility, and usability. Scientometrics, which began in the early 19th century and has gradually expanded since experienced a rapid shift at the beginning of the 21st century when the internet became accessible to all [5]. Furthermore, the usefulness of bibliometrics in the medical literature has increased 57-fold over the past 20 years [6]. The use of bibliometric methods in scientific and professional analysis has a more complex structure than simple listings of scientific productions or citation indexing [7]. Using statistical analyses of the increasing amount of data available makes it possible to reliably map scientific development, cooperation, and rankings [8]. Moreover, it provides authors with a highly accurate analysis of the amount of research in a specific country, specific subjects, and the impact of the specified research area. Furthermore, bibliometric analysis is an accurate and valid way to measure the impact of published articles [9,10].

Although this field has existed for many years, few bibliometric studies have focused primarily on dermatological literature [11-13]. Alsaif et al. [14] conducted a bibliometric analysis of dermatological publications in SA utilizing the PubMed database. Although PubMed is strongly associated with the medical field, it has some drawbacks. It is not considered a citation metrics database, and it has lower coverage of articles compared to other databases; thus, it does not represent the full publication pattern in SA [15,16]. On the other hand, Web of Science (WoS) and Scopus are the most commonly used search engines for bibliometric analysis. While WoS was the first citation indexing database, Scopus and Google Scholar were introduced later, in 2004 [17]. They also have larger scopes that include medical sciences, engineering, physics, and many others [18]. Although Google Scholar provides a higher citation count, it might include duplicate citations and articles that lack peer review, and its scope is unclear [19-21]. The WoS citation index is utilized to identify journals’ impact factors. This study reviewed citation-based research to evaluate previously published dermatology-related publications from SA using the WoS and Scopus databases.

## Review

Materials and methods

We conducted a retrospective, cross-sectional bibliometric analysis using the WoS and Scopus databases to retrieve all SA dermatology research from the databases’ respective dates of conception until July 9, 2021. As different databases provide different data, WoS and Scopus were both utilized in order to cover a larger number of SA-affiliated publications [22]. Ethical approval was not required since the present research assessed previously published studies.

The bibliographic analysis was conducted in three steps. First, in WoS advanced search, the field tags SU=dermatology and CU=Saudi Arabia were used to retrieve all SA-affiliated dermatology publications. The data were then exported into a Microsoft Excel sheet. Then, we repeated the same search strategy for all other Arab countries. A Scopus search was also performed, using SA as the affiliation country and dermatology as the source title, to retrieve all SA-affiliated dermatology publications. Microsoft Excel 2019 was used for data import and analysis. We combined all the SA articles and the journals in which they were published into one Excel sheet. The data combined included the title, year of publication, journal name, and digital object identifier (DOI) for each article. The missing data in the Excel sheet were obtained manually. If an article had different publication and early access dates, we included the publication date and excluded the early access date. However, if the article only had an early access date, we included it. Next, the "delete duplicate" function was used in the DOI column to ensure an accurate number of published articles. The number of citations and Hirsch indexes (h-indexes) of institutions and countries were obtained from WoS only. The numbers of publications from particular institutions and countries (including the number of citations) were retrieved from WoS only because this database provides both qualitative and quantitative data, such as the h-index and the number of citations. Population numbers were retrieved from the United Nations Population Division Estimates, 2021.

Results

**Figure 1:**
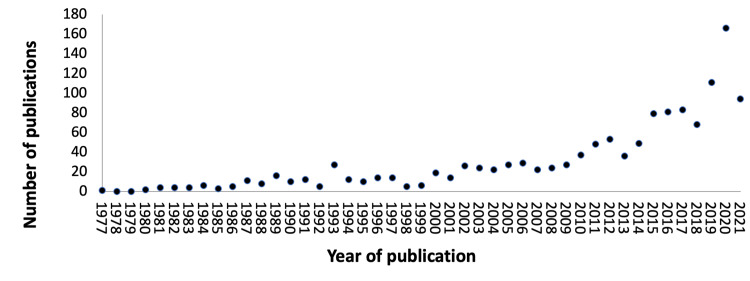
The number of new publications each year until July 9, 2021.

Saudi Arabia published 1,319 dermatology articles in WoS and Scopus. The number of published articles each year ranged from two to 14 in the 1980s and 1990s, respectively, with the exception of 1993, in which 27 articles were published. Approximately half (n=603) of Saudi-affiliated articles were published in the past six years (Figure 1).

**Figure 2:**
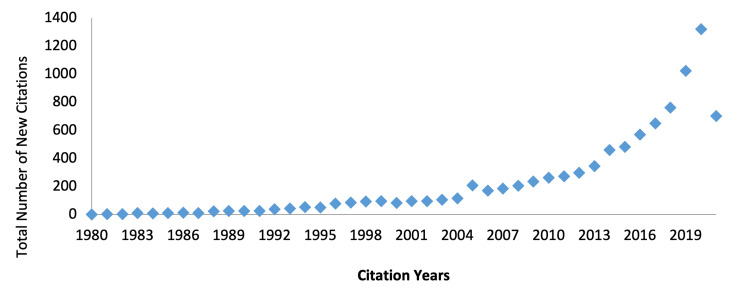
The number of citations each year, according to Web of Science.

According to WoS, the total number of citations was 9,285 (8,981 excluding self-citations). In 1980, the number of citations was 0; then, it increased gradually, to 115 in 2004, and to 569 in 2016 (Figure 2). More than half of all citations (n=5020, 54%) occurred in the past six years. The h-index, according to WoS, was 40 (Figure 2).

Table 1 represents Saudi institutions affiliated with 30 or more dermatology articles published from 1980 to 2020. Notably, eight of the 10 top institutions were universities. King Saud University (KSU) published 273 articles, representing approximately one-fifth (20.7%) of all published articles, and it had the highest h-index (29). It was followed by King Faisal University (KFU) and King Faisal Specialist Hospital & Research Centre, with 133 and 125 publications, respectively. King Saud bin Abdulaziz University for Health Sciences (KSAUHS) published 124 articles. However, KSAUHS is a relatively new institution; 49 of its articles were published in the past three years. KSU had the highest number of publications in one year (29 articles), followed by KFU (27 articles) and KSAUHS (25 articles).

**Table 1:** Saudi institutions with 30 or more articles, according to Web of Science.

Institution	H-Index	Number of publications (%)
King Saud University	29	273 (20.7%)
King Faisal University	17	133 (10.1%)
King Faisal Specialist Hospital & Research Centre	18	125 (9.5%)
King Saud bin Abdulaziz University for Health Sciences	15	124 (9.4%)
Imam Abdulrahman Bin Faisal University	16	91 (6.9%)
King Abdulaziz University	13	91 (6.9%)
Prince Sultan Military Medical City	16	62 (4.7%)
King Abdulaziz Medical City Riyadh	13	49 (3.7%)
King Khalid University Hospital	13	48 (3.6%)
Al Imam Muhammad Ibn Saud Islamic University	8	37 (2.8%)
Prince Sattam Bin Abdulaziz University	6	35 (2.7%)
Qassim University	6	33 (2.5%)

The highest number of published articles (n=144) was in the International Journal of Dermatology. The Journal of the American Academy of Dermatology had the highest impact factor (2.96). Only one of the top 10 journals was local. Further details are shown in Table 2.

**Table 2:** Top 10 journals publishing the highest numbers of SA-affiliated articles.

Journal	Publications	Impact factor
International Journal of Dermatology	144	0.72
Journal of the American Academy of Dermatology	109	2.96
Journal of Dermatology and Dermatologic Surgery	83	0.16
Burns	63	0.78
Clinical, Cosmetic and Investigational Dentistry	52	0.31
British Journal of Dermatology	43	2.45
Indian Journal of Dermatology, Venereology & Leprology	39	0.52
Journal of Cutaneous Medicine and Surgery	38	0.65
Journal of The European Academy of Dermatology and Venereology	34	1.71
Journal of Investigative Dermatology	32	2.51
Mycoses	32	1.45

Table 3 shows the publication frequencies of the most frequently encountered dermatological diseases in SA. Psoriasis was associated with the highest number of publications and citations in SA, followed by acne. Notably, inpatient dermatological diseases were associated with the lowest number of articles.

**Table 3:** The most common dermatological diseases in publications in the Web of Science Journal from Saudi Arabia.

Diseases	Publications	Citations
Psoriasis	198	2046
Acne	170	1852
Vitiligo	153	1438
Atopic dermatitis	120	1378
Hair Loss	115	1219
Lichen planus	80	760
Alopecia areata	51	554
Warts	50	346
Pemphigus vulgaris	28	310
Bullous pemphigoid	12	65
Tinea pedis	9	155
Pityriasis versicolor	7	48
Stevens-Johnson syndrome	2	0

The total number of Arab articles in WoS was 6,566. Egypt represented the largest percentage of publications (31.9%), followed by SA, representing 19.4%. H-indexes were highest for Egypt and SA (48 and 40, respectively). Libya had the highest average number of citations per article, followed by Syria and Kuwait. Lebanon and Kuwait had the highest numbers of publications per 1,000,000. More details are shown in Table 4.

**Table 4:** The number of publications and citations from Arab countries.

Country	Publications	H-index	Citations per article
Egypt	1966	48	8.24
Saudi Arabia	1194	40	7.78
Tunisia	837	31	5.92
Lebanon	558	32	9.96
Morocco	452	21	5.41
Kuwait	310	35	13.26
United Emirates	295	24	7.24
Jordan	170	22	12.75
Iraq	149	20	12.35
Algeria	146	16	6.89
Qatar	137	17	8.93
Libya	72	17	16.31
Bahrain	60	14	9.3
Sudan	55	14	11.6
Yemen	47	8	6.55
Oman	46	13	10.28
Syria	45	14	16.16
Palestine	27	9	12.07

Discussion

Our results included a total of 1,319 SA-affiliated dermatology articles published in WoS and Scopus, with nearly half of these published in the past six years. Moreover, citations increased dramatically during the past five years. Al-Aboud et al. [12] conducted a study utilizing scientometrics to assess the quality and quantity of dermatological studies in the Gulf region from 1966 to 2004. They found that dermatology publications witnessed a surge in the years 1990, 1992, and 1996, then increased gradually to reach their apex by 2002, then decreased again in 2003. Their study, however, did not provide h-indexes or numbers of citations per country, requiring further studies to be conducted.

The number of SA-affiliated publications has rapidly increased over the past five years. There are many potential reasons for this increase. First, the number of dermatologists has increased in the past five years, from 1,701 to 2,600, according to the Ministry of Health Statistical Yearbook [23]. Additionally, there have been a growing number of universities and increased funds for research centers in SA [1]. Furthermore, all academic doctors and medical students in Saudi Arabia have open access to almost all subscription-based journals and search engines, such as Scopus, through the Saudi Digital Library, which provides excellent research support [24,25]. Finally, the research requirement of the Saudi Commission for Health Specialties for acceptance into higher education programs, board certification, and promotion of academic doctors has also contributed to the rapid increase in SA-affiliated research.

Egypt was associated with a higher number of published dermatology papers than Saudi Arabia, and both were well behind many other countries. The highest number of publications was recorded in the USA, followed by Germany, England, Japan, and France. The USA's large number of studies may be explained by its large population compared to other countries [11]. However, the data should be normalized for the population. In our study, nine of the top 10 published articles were in international journals. However, in a study conducted by Alsaif et al. [14], two Saudi journals-Annals of Saudi Medicine and the Saudi Medical Journal-were among the top three. The difference in publication journals between their study and ours may be due to variations in the number of samples obtained between the two studies. Additionally, Saudi dermatologists are more willing to publish in journals with higher impact factors (i.e., international journals), even if publication is expensive [10, 24]. On the other hand, one study reporting trends in ophthalmology research in SA found that the Saudi Journal of Ophthalmology was the second-most common journal for publication among Saudi ophthalmologists [26].

Psoriasis was the most commonly investigated condition found in our study, followed by acne and vitiligo, and the least frequently studied conditions were inpatient dermatological diseases. A previous cross-sectional study reported the prevalence of vitiligo in SA to be approximately 3.5% [27]. Another meta-analysis reported it to be between 5.6% and 6.3% [28]. Vitiligo’s prevalence does not justify the increase in publications. However, the psychological impact of vitiligo in SA is high, leading to further investigation and additional published articles [29]. Though hair loss is more common than some other conditions, studies investigating it found it to be less frequent. A cross-sectional study reported the prevalence of hair loss to be 13.8%, which is more than double that of vitiligo [30]. This information indicates that we should be offering more support to research in favor of hair loss and other less popularized topics, such as inpatient dermatological diseases.

For institutions publishing the highest numbers of SA-affiliated articles (using 30 or more publications as a benchmark), King Saud University was at the top of the list, followed by King Faisal University, and in third place was King Faisal Specialist Hospital & Research Centre. An institution’s research significantly benefits its graduates in their careers; it has been postulated that future academic productivity could be predicted using medical school publications [31]. Therefore, we encourage institutes to further assist their students in conducting dermatology research by funding said research and offering materials and mentors.

One limitation of our study is that we only included articles from two search engines. This may limit our results by underrepresenting local journals, many of which are not indexed in WoS or Scopus. This factor sheds light on the importance of a local dermatology journal with a high impact factor that enriches the growth of dermatology research nationally.

## Conclusions

This bibliometric analysis revealed a dramatic increase in dermatology-related publications and citations in SA. These results shed light on the importance of investigating the tendency of Saudi researchers to publish in international journals and enhancing the quality of local journals. We encourage researchers to use the data presented herein to create a worldwide comparison of dermatology trends in different countries. Moreover, periodic bibliometric analysis can direct researchers and funding to areas in which research is lacking. The expansion of bibliometric analysis to other major specialties, such as medicine, surgery, and nursing, will help identify strengths and weaknesses in SA-affiliated research and achieve the goals of Saudi Vision 2030 to optimize health care and improve the quality of research in SA.
